# Rituximab monotherapy versus glucocorticoid therapy for adult minimal change disease: a retrospective study on noninferiority

**DOI:** 10.3389/fneph.2025.1715546

**Published:** 2025-12-09

**Authors:** Xiaoyun Li, Guoxiang Yao, Yujiao Sun, Na Li, Caifeng Gao, Haiping Wang, Rong Wang, Bing Chen

**Affiliations:** 1 Department of Nephrology, Shandong Provincial Hospital Affiliated to Shandong First Medical University, Jinan, Shandong, China; 2 Department of Nephrology, Jinan Zhangqiu District People’s Hospital, Jinan, Shandong, China; 3 Department of Nephrology, Jinan Shizhong District People’s Hospital, Jinan, Shandong, China

**Keywords:** nephrotic syndrome, minimal change disease, rituximab, glucocorticoid, induced remission

## Abstract

**Introduction:**

To verify whether rituximab (RTX) monotherapy is noninferior to glucocorticoids in inducing and maintaining remission in adults with minimal change disease (MCD).

**Method:**

We retrospectively analyzed the clinical data of 60 patients with minimal change disease (MCD) who were diagnosed with MCD by renal pathology biopsy and electron microscopy before their first visit to the Department of Nephrology of Shandong Provincial Hospital between 01/2020 and 01/2024, and were diagnosed with MCD at the first visit without acute kidney injury (AKI). Patients were divided into a RTX treatment group (RTX group, 20 cases) and glucocorticoids (GC) treatment group (GC group, 40 cases). None of the patients had previously received steroid/immunosuppressive therapy. The RTX group received rituximab monotherapy. At the 6-month follow-up, the RTX group received additional rituximab infusions as maintenance therapy. The primary endpoints were the time to induced remission, 12-month remission, and relapse rates in each group; the secondary endpoints were the safety and incidence of side effects.

**Results:**

After treatment during the 12-month follow-up period, 57 out of 60 patients (95%) achieved remission, of which 48 (80%) achieved complete remission; and 9 (15%) patients relapsed during the follow-up period. A total of 24 (40%) patients experienced adverse events while receiving treatment. 19 (95%) patients in the RTX group and 38 (95%) patients in the GC group achieved remission within 12 months of follow-up, respectively [the difference in rates between the two groups was 0%, 95% confidence interval (0.08, 11.73)]. In the RTX group, 14 (70%) achieved complete remission. In the GC group, 34 (85%) achieved complete remission (*p*=0.304). In the RTX group, 2 (10%) patients relapsed, and in the GC group 7 (18%) patients relapsed (*p*=0.701). 1 (5%) patient in the RTX group and 23 (58%) patients in the GC group experienced adverse events (*p*=0.000), none of which were severe.

**Conclusion:**

Adequate RTX monotherapy is noninferior to adequate glucocorticoids in inducing and maintaining remission in adult MCD patients without AKI, with fewer adverse effects and better adherence, and may be considered as a first-line treatment option for adult MCD patients without AKI.

## Introduction

Minimal change disease (MCD) is a common pathological type of nephrotic syndrome, affecting approximately 15% of adult patients with idiopathic nephrotic syndrome and an even higher proportion of pediatric patients (1). MCD manifests itself as nephrotic syndrome, which is defined as >3.5 g per day of proteinuria, leading to hypoproteinemia (<30 g/L), edema, and hyperlipidemia (2). No significant changes are observed under light microscopy, although the loss of pedunculated cell fusion is the only morphological change observed under electron microscopy (1). Adult-onset MCD may present severe clinical manifestations and lead to end-stage kidney disease (3).

Glucocorticoids (GC) remains the first-line treatment for MCD in adults (4). Some studies show that approximately 75% of patients with MCD achieve remission after glucocorticoid treatment, but approximately 56–76% of patients with MCD relapse after remission (3, 5). Long-term GC application can lead to the development of adverse effects such as dyslipidemia, impaired fasting glucose, osteoporosis, hypertension, and increased cardiovascular events (4). Recent studies have demonstrated that drugs such as tacrolimus, cyclophosphamide, and Mycophenolate mofetil can be used as alternative immunosuppressants to reduce GC dosage and are considered to be second- or third-line therapeutic options for patients with drug-resistant MCD, and some studies have even demonstrated that tacrolimus alone is effective in inducing an initial MCD remission and can be used as an alternative therapeutic option for patients with hormone-dependent MCD (1, 6, 7). Moreover, these drugs can effectively induce MCD remission, but their adverse effects and cytotoxicity limit their use in long-term maintenance therapy; therefore, safer and more effective therapeutic options need to be explored and researched.

MCD is a podocyte injury of uncertain etiology; it has been suggested that the glomerular filtration barrier is impaired in MCD, primarily due to the loss of slit diaphragm tissue, of which nephrin is an important component (8). MCD is an autoimmune disease, and that auto-antibodies against nephrin may interfere with the integrity of the slit diaphragm complex, leading to massive proteinuria, an idea supported by the reduction or complete disappearance of circulating auto-nephrin antibodies during the response to treatment (9). In addition, B and T cells are involved in the pathogenesis of MCD and that the active phase of MCD is associated with an increase in the number of T and B lymphocytes, as well as an increase in cytokine production (10). Recently, increased expression of CD80 (also known as *B7-1*) on podocytes has been identified as a mechanism for proteinuria. CD80 is a costimulatory factor expressed by antigen-presenting cells and B-cells, and it is inhibited by binding to CTLA-4 expressed on regulatory T-cells. Interleukin-13, or microbial products via Toll-like receptors, may be factors that induce CD80 expression in podocytes expression (11, 12).

Rituximab (RTX) is a chimeric human immunoglobulin G1 anti-CD20 monoclonal antibody that specifically binds to depleted CD20-positive B cells (13). RTX has also been shown to modulate the activity of acid-sphingomyelinase (ASMase), which is essential for signaling molecules in podocytes, and RTX may protect the glomerular filtration barrier at the source by preventing actin cytoskeleton remodeling in podocytes by preserving sphingolipid-associated enzymes as well as SMPDL-3b and ASMase activities (14, 15). In addition, RTX was extremely well tolerated in the studies published to date, with a rate of 0.092 serious adverse events per year, and most of the adverse events were associated with the administration of higher doses of RTX (7). In recent years, RTX has been approved for use in various autoimmune and renal diseases, such as membranous nephropathy and ANCA-associated vasculitis (16, 17). RTX injection from Fosun Pharmaceuticals was chosen as the primary drug of interest in this study, and this anti-CD20 monoclonal antibody has been studied for equivalence with imported RTX from Roche Diagnostics Gmbh (18).

Few studies report of direct induction of remission of MCD in adults with full dose of RTX alone in China and abroad; thus, this study describes how a full dose of RTX alone in first induction of MCD remission in adults with MCD can help improve the outcome of MCD. The efficacy and safety of RTX alone for the initial induction of remission in patients with MCD and whether there was any significant difference between RTX and conventional treatment regimens of GC or GC combined with tacrolimus were analyzed. This provides new protocols and ideas for the induction and maintenance of MCD remission.

## Materials and methods

### Research target

**Figure 1:**
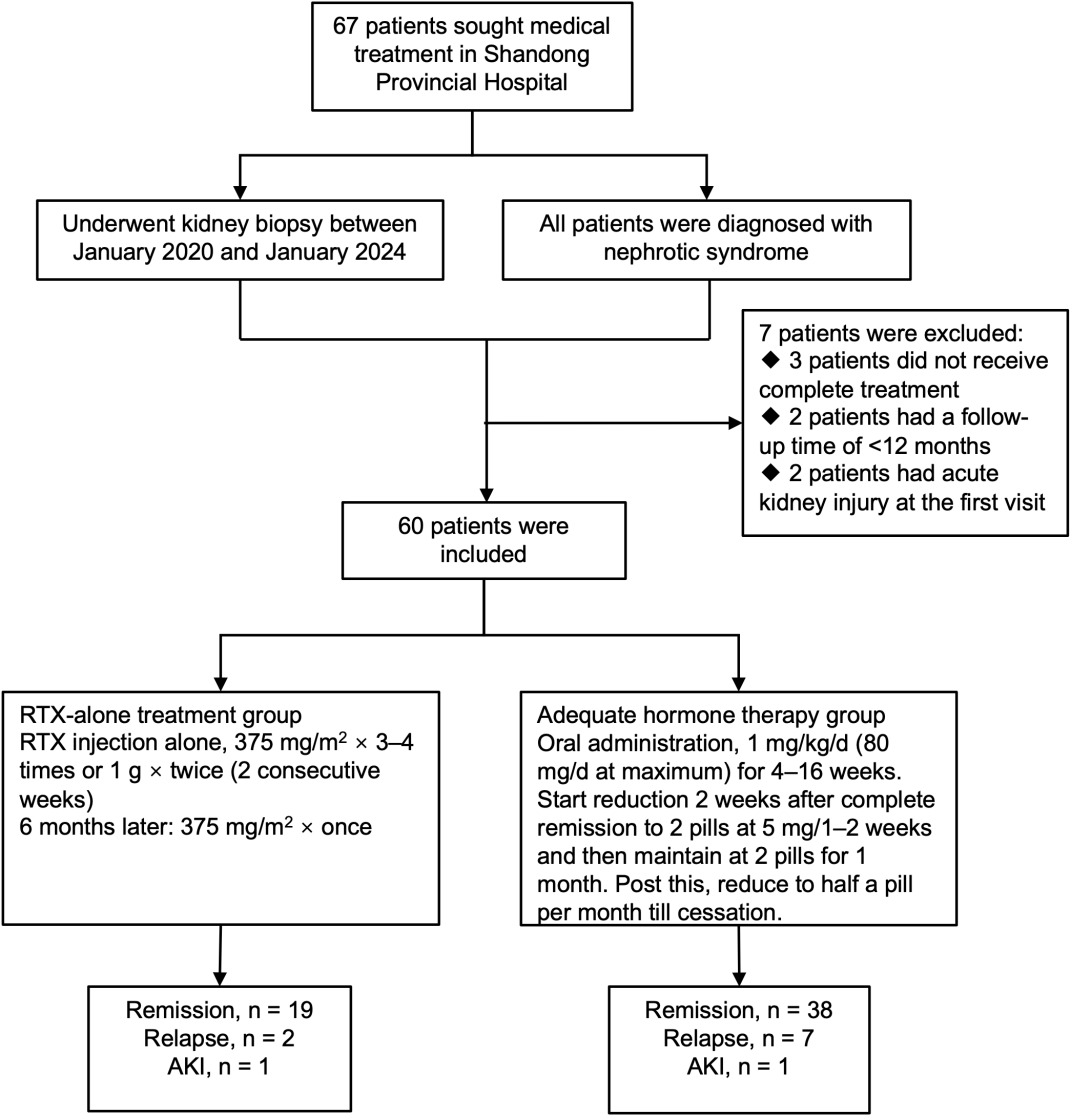
Flowchart of patients with minimal change disease receiving different treatment regimens. AKI, acute kidney injury; RTX, rituximab.

Sixty adult patients with MCD who visited the Nephrology Department of Shandong Provincial Hospital between 01/2020 and 01/2024 were included in this study. All patients had previously undergone renal biopsy with electron microscopy to confirm the diagnosis of MCD, and only those patients who were not associated with acute kidney injury (AKI) at the first visit were included. The inclusion criteria were as follows: (1) all patients had clinical manifestations of nephrotic syndrome with 24-h urine protein > 3.5 g/24 h and serum albumin<30 g/L (1); (2) all patients had a confirmed diagnosis of MCD by renal biopsy with electron microscopy prior to the initial treatment; (3) all patients had well-documented clinical and laboratory examinations; and (4) The patient underwent a thorough evaluation to rule out potential causes of secondary MCD, including a detailed medical history review, physical examination to assess for underlying tumors, review of potentially causative medications, screening for hepatitis B and C viruses, and antinuclear antibody testing. The exclusion criteria were as follows: (1) patients with poor medical compliance and <1 year of follow-up, (2) patients who did not receive complete treatment owing to their condition, (3) patients with AKI before the initial consultation. 60 patients were enrolled in this study and divided into two groups of 1:2 according to the different initial treatment regimens received by the patients. The first group of patients was the rituximab monotherapy group (RTX group, n=20), and the patients in this group received the RTX alone at 375 mg/m^2^ × 3–4 times or 1 g × 2 times (2 weeks); the second group of patients was the adequate Glucocorticoid therapy group (GC group, n=40), in which the patients were directly treated with an adequate amount of GC (1 mg/kg/d) as the first treatment regimen. A noninferiority test was performed on the remission rates of the RTX and GC groups, assuming a noninferiority cutoff value (Δ) of 0.1. 20 patients in the RTX group and 40 in the GC group were included in the study (**Figure 1**). This study complied with the Declaration of Helsinki and was approved by the Ethics Committee of Shandong Provincial Hospital (SWYX: NO. 2023-310).

### Clinical information

Basic information included sex, age, and time of onset. Laboratory tests include urine protein quantification, urine routine, serum total protein, serum albumin, total cholesterol, serum creatinine, urea nitrogen, glomerular filtration rate, hemoglobin, white blood cell count, platelet count, lymphocyte count, CD19 “B cell count, CD20 “B cell count, immunoglobulin G (IgG), immunoglobulin A (IgA), immunoglobulin M (IgM), etc. The follow-up data included follow-up time, remission, number of relapses, and adverse reactions.

### Treatment

None of the patients had previously received steroid/immunosuppressive therapy. All are first-time patients. The RTX group received rituximab monotherapy, without previously, concurrently, or subsequently administered corticosteroid therapy. Two dosing regimens of RTX were formed in this study: RTX intravenously, 375 mg/m^2^ at a time, once a week for 4 weeks as a course of treatment; and RTX intravenously, 1 g/dose, to be used at 2-week intervals, with a total of two uses as a course of treatment. Both regimens have been shown to have the same effect in other diseases, with no statistically significant difference (19). Patients in the RTX treatment group who achieved remission will be evaluated at 6 months post-treatment based on their B-cell rebound levels and remission status to determine eligibility for a single 375 mg/m² re-injection. Specific doses were administered as described above. The dosing regimen in the adequate GC group was oral administration of 1 mg/kg/d (maximum 80 mg/d) for a minimum of 4 weeks and a maximum of 16 weeks. GC tapering was initiated after 2 weeks of complete remission, with the GC dosage reduced to two tablets at a rate of 5 mg/1–2 weeks, followed by two tablets maintained for 1 month, and then reduced by half a tablet per month until tapering was discontinued, for a total GC exposure time of approximately 36–48 weeks. All patients were followed up in the study at week 2 and at 1, 2, 3, 6, 9, and 12 months after treatment, with routine blood, urine, liver function, biochemistry, 24-h urine protein quantification, and circulating B-cell counts. Complications and relapses were observed and recorded, and adverse events during drug treatment and throughout the follow-up period were recorded. The primary endpoints of this study were the time to induction of remission and the 12-month remission and relapse rates of patients in each group, while the secondary endpoints were safety and incidence of side effects.

### Definition of indicators

1. Mitigation time

Time of first dose to time of urine protein conversion.

2. Complete remission (CR)

Quantitative 24-h urine protein decreased to less than 0.3 g/d or urine protein/creatinine (uPCR) <300 mg/g (or <30 mg/mmol); stable creatinine and serum albumin >3.5 g/dL (or 35 g/L).

3. Partial remission (PR)

Decrease in 24-h urine protein quantification to less than 0.3–3.5 g/d or uPCR<300–3500 mg/g (or <30–350 mg/mmol); or 50% decrease in 24-h urine protein quantification from baseline levels and stable renal function (<20% increase in blood creatinine from baseline levels).

4. Relapse

Patients in remission after treatment reappear with a 24-h urine protein quantification > 3.5 g or uPCR > 3,500 mg/g.

5. No remission (NR)

Massive proteinuria (>3.5 g/d), an insignificant decrease in urinary protein (<50% decrease from baseline value), and/or a significant increase in Scr (increase in Scr > 50% of baseline value).

6. Acute kidney injury (AKI)

Elevated creatinine ≥ 0.3 mg/dL within 48 h, or creatinine ≥ 50% above basal value within 7 d, or decreased urine output [<0.5 mL/(kg·h)), lasting ≥6 h].

7. Deterioration of kidney condition

Post-treatment rise in serum creatinine > 133 μmol/L or doubling of baseline serum creatinine level lasting > 3 months.

8. End-stage renal disease (ESRD)

Creatinine clearance<15 mL/min at the last follow-up, dialysis initiation, or renal transplantation.

9. Adverse reactions

Vegetable-related adverse reactions include osteoporosis, infections, gastrointestinal bleeding, electrolyte disturbances, hypertension, hyperglycemia, cataracts, weight gain, induced grand mal seizures, and psychiatric symptoms. The most common adverse reactions to RTX are infusion-related adverse reactions including rash, erythema, itching, runny nose, and irritability (20, 21).

10. Serious adverse events

Serious adverse events include clinical death or the occurrence of a life-threatening disease such as severe pulmonary infection, pulmonary embolism, cerebral infarction, myocardial infarction, or an adverse event resulting in hospitalization of the patient (20, 21).

### Statistical methods

Statistical analyses were performed using SPSS 26.0. Continuous variables that conformed to normal distribution were expressed as mean ± standard deviation, and t-test was used for comparison between two data groups. Noncontinuous variables that did not conform to a normal distribution are expressed as medians (interquartile ranges), and comparisons between the two data groups were made using the rank sum test. One-way ANOVA was used for between-group comparisons of multiple (≥3) continuous variables. Categorical variables were expressed as frequencies, and all comparisons between groups were made using the χ^2^ test. The probability of the noninferiority test was one-tailed and the statistical significance was set at *p*<0.025, whereas the probability of the rest of the tests was two-tailed and the statistical significance was set at *p*<0.05, with *p*<0.01 indicating a highly significant difference.

## Results

### Baseline information

Sixty patients with an initial diagnosis of MCD were included in this study, with a median age of 34.00 (23.00, 52.00) years, of which 33 were male and 27 were female. The patients were all first-time patients, and before performing the consultation, the median level of 24-h urine protein quantification in all patients was 7.43 (6.31,9.72) g/24 h, the median level of serum albumin was (18.90 (16.18,22.10)) g/L, the mean level of serum creatinine was 69.80 ± 18.27 µmol/L, and the mean level of eGFR was 110.79 ± 24.94 mL/min/1.73 m^2^ (**Table 1**).

**Table 1:** Baseline characteristics of 60 adult patients with minimal change disease included in this study.

Characteristic	Total (n=60)	RTX group (n=20)	GC group (n=40)	*p*
Sex (male), n (%)	33 (55.00)	12 (60.00)	21 (52.50)	0.58
Age (years)	34.00 (23.00, 52.00)	34.00 (25.00, 43.25)	33.50 (22.25, 59.00)	0.96
Urine red blood cells (/ul)	1.85 (0.68, 4.93)	1.50 (0.40, 5.35)	2.40 (0.70, 4.20)	0.53
Urine protein (g/24 h)	7.43 (6.31, 9.72)	7.08 (6.45, 8.99)	7.62 (6.30, 10.37)	0.47
Hemoglobin (g/L)	143.17 ± 20.76	141.68 ± 23.66	143.88 ± 19.51	0.71
White blood cells (×10^9^/L)	5.69 (4.65, 6.82)	5.36 (4.70, 6.48)	5.91 (4.54, 7.09)	0.62
Platelet (×10^9^/L)	261.50 (237.50, 321.00)	250.50 (227.25, 305.75)	266.50 (242.75, 336.00)	0.23
Aspartate aminotransferase (u/L)	27.50 (21.00, 35.00)	25.00 (19.50, 31.00)	29.00 (22.25, 39.50)	0.12
Alanine aminotransferase (u/L)	23.00 (14.25, 36.00)	23.00 (15.25, 32.00)	22.00 (13.25, 37.50)	0.97
Total cholesterol (mmol/L)	10.60 ± 3.69	10.25 ± 3.70	10.74 ± 3.72	0.65
Serum total protein (g/L)	42.62 ± 5.87	43.33 ± 5.88	42.28 ± 5.90	0.53
Serum albumin (g/L)	18.90 (16.18, 22.10)	19.85 (16.78, 25.38)	17.25 (15.45, 21.43)	0.09
Serum globulin (g/L)	23.20 (20.80, 25.58)	22.80 (20.85, 23.68)	23.20 (20.30, 26.38)	0.62
Blood urea nitrogen (mmol/L)	4.80 (4.25, 5.70)	4.80 (4.40, 5.40)	4.85 (3.80, 7.10)	0.80
Serum creatinine (μmol/L)	69.80 ± 18.27	69.80 ± 20.23	69.80 ± 17.48	1.00
eGFR (mL/min/1.73 m^2^)	110.79 ± 24.94	114.31 ± 20.20	108.89 ± 27.24	0.44
Lymphocyte count (×10^9^/L)	1.86 (1.46, 2.31)	1.77 (1.36, 2.18)	1.91 (1.50, 2.34)	0.40
CD19+ B-cell count (/ul)	249.56 (188.55, 290.86)	283.51 (213.94, 360.14)	206.28 (132.33, 277.24)	0.10

eGFR, estimated glomerular filtration rate; GC, glucocorticoid; RTX, rituximab.

### Assessment of efficacy

**Figure 2:**
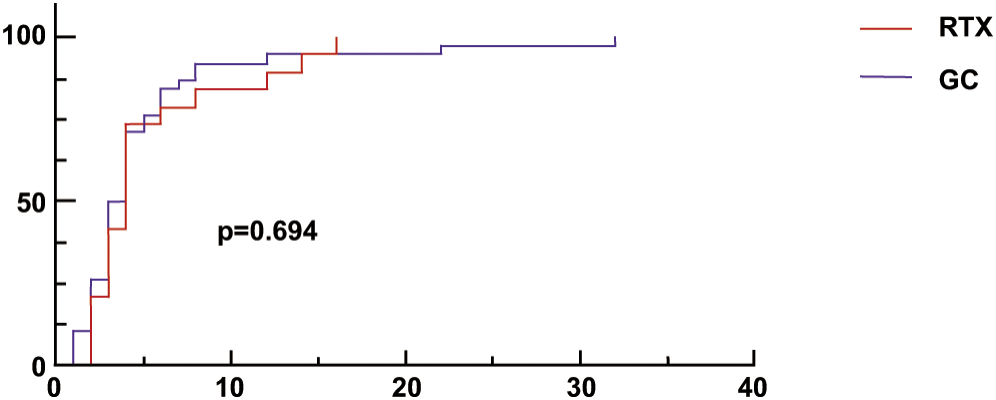
Trends in the remission rates during the 12-month follow-up period. GC, glucocorticoid; RTX, rituximab.

Sixty adult patients with MCD were followed up for 12 months. At the 12th month of completion of treatment, 57 (95%) patients achieved remission, of which 48 (80%) achieved complete remission, 9 (15%) achieved partial remission with a median time to remission of 4 (2.5, 5.5) weeks, and 9 (15%) patients relapsed. In the RTX group, 19 patients (95%) achieved remission, 14 (70%) achieved complete remission, 5 (25%) achieved partial remission with a median time to remission of 4 (3, 6) weeks, 2 (10%) patients relapsed, and 9 (45%) patients were given one dose of RTX 375 mg/m^2^ at 6 months after completion of the treatment with full dose of RTX. Thirty-eight (95%) patients in the GC group achieved remission, 34 (85%) achieved complete remission, and 4 (10%) achieved partial remission, with a median time to remission of 3.5 (2, 5.25) weeks, and 7 (18%) patients relapsed (**Table 2**). remission rate in the RTX group was 95%, and in the GC group, with a noninferiority cutoff of Δ=0.1, the difference between the rates of the two groups was 0%, 95% CI (0.08, 11.73), and the confidence interval (0.08, 11.73) was completely higher than -0.1; thus, the effectiveness of the RTX group can be considered noninferior to the GC group. During the study follow-up period, the remission rate of all patients gradually increased with the prolongation of follow-up time (**Figure 2**), and the level of remission rate was consistent between the RTX group and GC group [95% vs. 95%, *p* =1.000], with no statistically significant difference between the groups. The level of relapse rate in the RTX group was lower than that in the GC group [10% vs. 18%, *p* =0.701], and the difference between the groups was also not statistically significant.

**Table 2:** Remission and relapse in patients.

Characteristic	Total (n=60)	RTX group (n=20)	GC group (n=40)	*p*
Time to remission (weeks)	4 (2.5, 5.5)	4 (3, 6)	3.5 (2, 5.25)	0.54
Remission rate, n (%)	57 (95.00)	19 (95.00)	38 (95.0)	1.00
Complete remission rate, n (%)	48 (80.00)	14 (70.00)	34 (85.00)	0.30
Partial remission rate, n (%)	9 (15.00)	5 (25.00)	4 (10.00)	0.25
Relapse rate, n (%)	9 (15.00)	2 (10.00)	7 (18.00)	0.70

Values are expressed as number (%), median (interquartile range), or mean ± standard deviation. GC, glucocorticoid; RTX, rituximab.

### Comparison of laboratory indicators themselves before and after treatment

**Figure 3:**
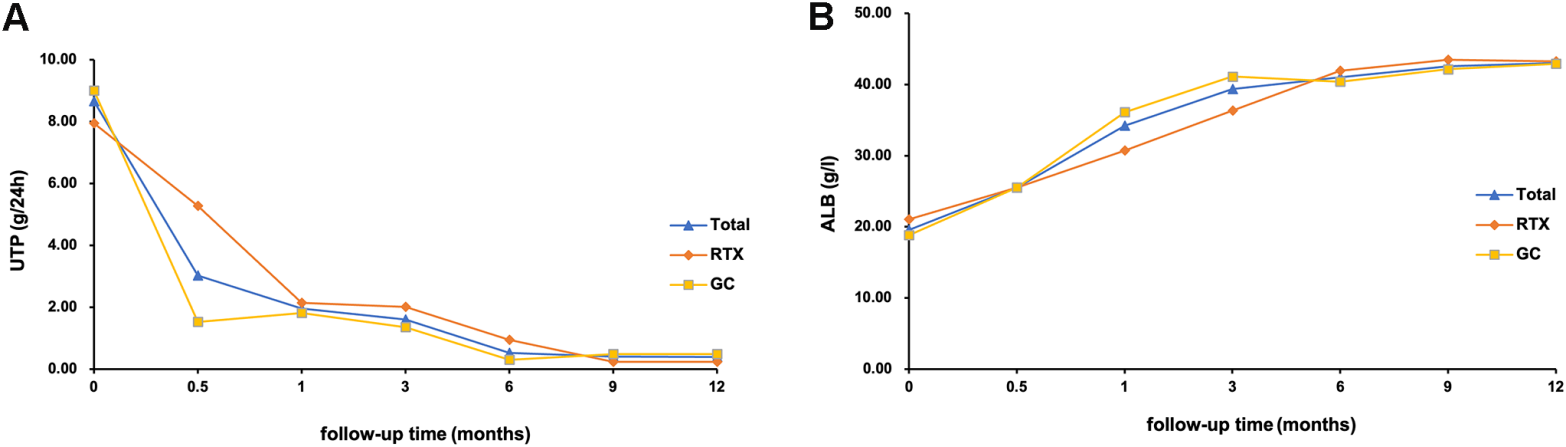
Trends in **(B)** albumin and **(A)** 24-hour urinary protein levels after treatment in patients who were followed up for 12 months. GC, glucocorticoid; RTX, rituximab; UTP, 24-Hour Urine Protein Quantification; ALB, Albumin.

During the 12-month follow-up period, quantitative urine protein levels decreased significantly and albumin levels increased significantly in all patients after 1 year of treatment. The 24-h urine protein level of all MCD patients decreased from 7.43 (6.31, 9.72) g/24 h to 0.08 (0.04, 0.15) g/24 h, and the difference between the two groups was not statistically significant after treatment (*p*=0.211); serum albumin gradually increased from 18.90 (16.18, 22.10) g/L to 43.50 (39.28, 46.55) g/L, and the difference between the two groups was also not statistically significant (*p*=0.690) (**Table 3**). During the 12-month follow-up period of the patients in the RTX group, the quantitative urinary protein and the absolute value of CD19 showed an overall decreasing trend, the serum albumin showed an increasing trend; the 24-h urinary protein level decreased from 7.08 (6.45, 8.99) g/24 h to 0.09 (0.06, 0.20)g/24 h and the absolute CD19 cell value decreased from 283.51 (213.94, 360.14)/µL to 23.71 (0.79, 103.79)/µL, whereas the serum albumin gradually increased from 19.85 (16.78, 25.38) g/L to 43.60 (38.50, 45.60) g/L (**Figure 3**).

**Table 3:** Clinical characteristics of MCD patients after rituximab treatment for 12 months.

Characteristic	Total (n=60)	RTX group (n=20)	GC group (n=40)	*p*
Urine protein (g/24 h)	0.08 (0.04, 0.15)	0.09 (0.06, 0.20)	0.07 (0.03, 0.14)	0.21
Serum albumin (g/L)	43.50 (39.28, 46.55)	43.60 (38.50, 45.60)	43.40 (39.30, 46.70)	0.69
Serum creatinine (μmol/L)	63.92 ± 12.42	60.77 ± 11.37	65.30 ± 12.85	0.39
Lymphocyte count (×10^9^/L)	1.56 (1.23, 2.12)	1.34 (1.12, 1.36)	1.80 (1.32, 2.18)	0.03
CD19+ B-cell count (/ul)	23.71 (0.79, 103.79)	23.71 (0.79, 103.79)		

Values are expressed as number (%), median (interquartile range), or mean ± standard deviation. One-way analysis of variance was performed between the groups, and no statistically significant differences (*p* > 0.05) were observed. GC, glucocorticoid; RTX, rituximab.

### Security analysis

A total of 24 cases (40%) experienced adverse events during the follow-up period. One patient (5%) in the RTX group developed itchy skin and a red rash after RTX titration, and the allergic symptoms were relieved by suspending RTX titration and administering antiallergic medication. Twenty-three cases (58%) in the GC group experienced adverse reactions, which manifested themselves as the most common forms of pharmacogenetic Cushing’s syndrome, obesity, hypertension, diabetes, osteoporosis, agitation, and other hormone-related adverse reactions. The incidence of adverse events was lower in the RTX group than in the GC group [5% vs. 58%, p=0.000], with a statistically significant difference between groups, which may be related to the small sample size (**Table 4**). The most common adverse reactions associated with RTX are infusion-related adverse reactions, which manifest as rashes, erythema, itching, runny nose, and irritability. These can be alleviated by increasing the means of pretreatment, such as increasing the dosage of steroids and combined application of Promethazine (20, 21).

**Table 4:** Adverse events in all patients with minimal change disease receiving treatment.

Event	Total (n)	RTX Group	GC Group
Total adverse events	24	1	23
Serious adverse events	0	0	0
Fatal	0	0	0
Non-fatal	24	1	23
Pulmonary infection	0	0	0
Non-serious adverse events	24	1	23
Infusion reaction*	1	1	0
Allergic rash	1	1	0
Hormone-related adverse reactions	23	0	23
Weight gain	6	0	6
Dyslipidemia	1	0	1
Abnormal blood glucose	2	0	2
Osteoporosis	1	0	1
Stretch marks (striae)	2	0	2
Headache	1	0	1
Depression	1	0	1
Acne	3	0	3
Agitation; insomnia	4	0	4
Hypertension	1	0	1
Gastrointestinal discomfort	1	0	1

GC, glucocorticoid; RTX, rituximab.

## Discussion

Currently, the first-line treatment of MCD is still based on the use of adequate amounts of corticosteroids and immunosuppressive drugs acting on different pathways of the immune system, such as cyclophosphamide, azathioprine, cyclosporine, tacrolimus, and Mycophenolate Mofetil (MMF), of which the steroid regimen is the most common; however, all of these drugs are associated with varying degrees of therapeutic toxicity and have many adverse effects (3). Thus, more rational and safer treatment options are yet to be explored. The emergence of RTX has addressed these issues. RTX was initially approved for the treatment of CD20-positive lymphomas, rheumatoid arthritis, and ANCA-associated vasculitis (22), and has since become increasingly widely used in various immune renal disorders, with guidelines suggesting that RTX is the preferred treatment for intermediate- to high-risk membranous nephropathy (23). In steroid-sensitive patients, RTX has been shown to be effective in reducing the dose of steroids and other immunosuppressive agents and is considered an effective and safe treatment option for the majority of adult patients with hormone-dependent or frequently relapsing MCD, although approximately 27% of patients may relapse (24, 25). Some studies have reported that RTX appears to be effective as a first-line therapeutic agent for MCD (26, 27), but no study has yet conducted a randomized controlled trial of adult patients with MCD to compare RTX therapy alone with currently used agents such as glucocorticoids, tacrolimus, cyclosporine, and MMF. In this retrospective study, we found for the first time that RTX monoclonal antibody alone in adequate doses was noninferior to hormonal therapy in inducing remission of proteinuria in adults with MCD, but RTX had fewer adverse effects and a better safety profile than steroids. RTX is an effective and safe therapeutic option for adult patients with MCD in the first stage of treatment, with promising clinical outcomes.

All patients enrolled in this study were first-time visitors who had not previously received treatment with steroids or immunosuppressive agents. Both groups received monotherapy without combination with other immunosuppressive agents. First, this study showed that 19 patients (95%) in the RTX group achieved remission. The remission rate in the RTX group was consistent with that in the GC group [95% vs. 95%, *p*=1.000], with no statistically significant difference between the groups; 14 patients (70%) achieved complete remission, and the complete remission rate in the RTX group was lower than that in the GC group [70% vs. 85%, *p* =0.304], and the difference between groups was not statistically significant. At 12 months of follow-up, the remission rate was 95% in the RTX group and 95% in the GC group, setting the noninferiority cutoff value Δ= 0.1. The difference between the rates of the two groups was 0%, with a 95% CI (0.08, 11.73) and a confidence interval (0.08, 11.73) of fully above -0.1. Thus, that it can be assumed that the effectiveness of the RTX group was noninferior to that of the GC group, demonstrating the effectiveness of RTX monotherapy in treating adult MCD. This result is superior to the remission rate of 93% (13 patients) in 14 MCD patients treated with a standard dose of RTX reported by Chang Wang (28) and superior to the remission rate of 78% (7 patients) in 9 adult-onset MCD patients reported by Nan Guan (29). This is also higher than the 76% and 74% remission rates reported in previous studies on daily and alternate-day steroid therapy, respectively (3). A recent study demonstrated that among 9 newly diagnosed adult MCD patients treated with rituximab monotherapy for 12 months, 8 patients (89%) achieved complete remission. This indicates that RTX monotherapy may be an effective treatment for newly diagnosed adult MCD patients, consistent with the conclusions of this study (30). Only one patient (5%) in the RTX group did not achieve remission within the 1-year follow-up time after completion of the full dose of RTX injections and gained remission with the addition of Obinutuzumab 1.0 g after 1 year. We suspected that this patient’s prolonged and massive proteinuria had led to chronic kidney injury, although the eventual development of focal segmental glomerulosclerosis (FSGS) is also more likely. Considering the reality of the patient’s situation and disease status, a renal puncture pathological biopsy was not performed again; the exact reason for this is unclear. Most patients unresponsive to rituximab develop advanced renal impairment, whereas responders largely retain renal function. Renal insufficiency correlates with treatment resistance and is commonly observed in patients with non-selective proteinuria. For those unresponsive to rituximab, the proteinuria selectivity index (PSI) can be assessed. This index not only predicts steroid response in adults but also identifies rituximab responders, thereby preventing unnecessary treatment in individuals unlikely to benefit (31). Overall, the results of our study were consistent with the findings of previous studies; however, the remission rates differed between studies, which may be explained by the inclusion criteria and the small sample size that may be subject to sampling error. The results of this study demonstrate that the efficacy of RTX alone is noninferior to that of an adequate hormone therapy regimen, and that RTX may be considered an alternative treatment option for adult patients with MCD.

Second, the present study concluded that RTX alone may be superior to steroids in maintaining long-term remission in adult MCD patients, and previous studies have demonstrated that more than one-third of hormone-sensitive MCD patients relapse after remission (3, 32, 33). During the follow-up period of 60 patients in this study, only 9 (15%) patients relapsed: 2 (10%) patients in the RTX group and 7 (18%) patients in the GC group. There was no significant difference in recurrence rates between the RTX group and the GC group [10% vs. 18%, *p* =0.701], Once again demonstrating that rituximab monotherapy is non-inferior to corticosteroid therapy in treating adult MCD. A retrospective study conducted across 30 nephrology departments in 15 countries worldwide demonstrated that rituximab promotes initial and long-term remission in most difficult-to-treat adult patients with MCD. Following rituximab treatment, a significant reduction in annualized relapse rates and the need for concomitant immunosuppression was observed (34). Although RTX itself is more costly, hormonal treatment regimens have a higher rate of relapses and more associated adverse events than those of the RTX regimen; considering the subsequent maintenance therapy plus treatment of adverse events, the total costs incurred by hormonal treatment may exceed those of RTX.

In terms of adverse events, only one patient (5%) in the RTX group developed itchy skin and red rash after the RTX drip, and the allergic symptoms were relieved by suspending the RTX drip and administering anti-allergic drugs. On the other hand, 23 patients (58%) in the GC group developed adverse reactions, which manifested as the most common hormone-related adverse reactions, such as pharmacogenetic Cushing’s syndrome, obesity, elevated blood glucose, osteoporosis, purple lines on the skin, acne, headache, agitation, mood loss, etc. The study results demonstrated a significantly lower incidence of adverse reactions in the RTX group compared to the GC group [5% vs. 58%, p=0.000], with a statistically significant difference between groups. This discrepancy may stem from the total glucocorticoid (GC) exposure duration in this study (36–48 weeks), which exceeded the maximum recommended duration of 25 weeks as per KDIGO guidelines. Prolonged GC use may increase the incidence of steroids-related adverse reactions and it may even lead to an elevated 12-month remission rate in the GC group. However, it also demonstrates that rituximab monotherapy in adult MCD is non-inferior to corticosteroid therapy in terms of safety. Most of the patients in this study tolerated RTX well, and 24 (40%) patients experienced adverse events during the follow-up period of receiving treatment, all of which were nonmalignant or fatal. These outcomes were less than the side effects previously reported with hormone therapy and RTX alone (6, 35, 36). This difference may be due to the fact that this study was a retrospective study, which resulted in some minor adverse events being missed. Other studies have reported that the incidence of serious adverse events can range from 0%–17%, with the most common adverse reaction being infusion reactions, which can be mitigated by modification of the pretreatment regimen (20, 21). Infusion of RTX does not affect renal function, with all renal function-related parameters being within the normal range at follow-up (28). The relatively low number of infusion reactions in this study is presumed to be related to the use of anti-allergic medication prior to infusion and limiting the rate of infusion to avoid or ameliorate infusion-related events. The safety of RTX for the treatment of MCD has been well established in this study.

Finally, the RTX regimen has an economic advantage when combined with the cost of the initial treatment of the patient and the treatment of associated adverse effects. Although consolidation therapy appears to increase the cost of treatment, The relapse rate in the RTX group was comparable to that in the GC group, fewer adverse effects, a higher quality of survival, and better protection of renal function. One study compared treatment costs before and after RTX therapy and found that overall costs may be lower because of longer remission periods (36). In addition, compliance with intravenous RTX is better when administered intermittently compared with daily oral hormone therapy; therefore, we recommends RTX as a first-line treatment option for adult patients with MCD rather than as an alternative treatment option, both from the point of view of efficacy and economy.

Our study is a retrospective observational study based on real-world data, which carries certain limitations and experimental biases. First, as a single-center retrospective study, it is subject to selection bias. The enrolled patients do not represent the entire target population, as the study only included cases from one top-tier hospital. Furthermore, the existing research data were derived from historical medical records, databases, or archives rather than being specifically collected to address the study’s research questions. Consequently, data may be incomplete or inaccurate, and these patients may have more severe conditions. Due to the presence of confounding factors, the findings may only provide preliminary non-inferiority evidence, necessitating further prospective studies for validation. Second, sample sizes were small. Although retrospective studies can establish control groups, selection for these groups may also be biased. Baseline characteristics between patient groups may lack comparability, unlike randomized controlled trials that balance known and unknown confounding factors through random assignment, preventing comprehensive explanation of all issues. Despite these limitations, our study lays the groundwork for future large-scale prospective research to further investigate the application of RTX in minimal change disease.

The efficacy of RTX monotherapy in inducing remission in adult patients with MCD without AKI is non-inferior to steroids, with fewer adverse reactions, and better adherence than steroidal agents. It may be considered as a first-line treatment option for adult patients with MCD without AKI.

## Data Availability

The datasets presented in this study can be found in online repositories. The names of the repository/repositories and accession number(s) can be found below: The data supporting the findings of this study are openly available in figshare at DOI:10.6084/m9.figshare.28129535.
